# Metabolic Implications of Immune Checkpoint Proteins in Cancer

**DOI:** 10.3390/cells11010179

**Published:** 2022-01-05

**Authors:** Elizabeth R. Stirling, Steven M. Bronson, Jessica D. Mackert, Katherine L. Cook, Pierre L. Triozzi, David R. Soto-Pantoja

**Affiliations:** 1 Department of Cancer Biology, Wake Forest School of Medicine, Winston-Salem, NC 27157, USA; estirlin@wakehealth.edu (E.R.S.); klcook@wakehealth.edu (K.L.C.); ptriozzi@wakehealth.edu (P.L.T.); 2 Department of Pathology, Section of Comparative Medicine, Wake Forest School of Medicine, Winston-Salem, NC 27157, USA; sbronson@wakehealth.edu; 3 Department of Internal Medicine, Section of Molecular Medicine, Wake Forest School of Medicine, Winston-Salem, NC 27157, USA; 4 Department of Surgery, Wake Forest School of Medicine, Winston-Salem, NC 27157, USA; jmackert@wakehealth.edu; 5 Wake Forest School of Medicine Comprehensive Cancer Center, Winston-Salem, NC 27157, USA; 6 Department of Hematology and Oncology, Wake Forest School of Medicine Comprehensive Cancer Center, Winston-Salem, NC 27157, USA; 7 Department of Radiation Oncology, Wake Forest School of Medicine, Winston-Salem, NC 27157, USA

**Keywords:** immunometabolism, immune checkpoint blockade, metabolism, bioenergetics, diet, immune-related adverse events

## Abstract

Expression of immune checkpoint proteins restrict immunosurveillance in the tumor microenvironment; thus, FDA-approved checkpoint inhibitor drugs, specifically PD-1/PD-L1 and CTLA-4 inhibitors, promote a cytotoxic antitumor immune response. Aside from inflammatory signaling, immune checkpoint proteins invoke metabolic reprogramming that affects immune cell function, autonomous cancer cell bioenergetics, and patient response. Therefore, this review will focus on the metabolic alterations in immune and cancer cells regulated by currently approved immune checkpoint target proteins and the effect of costimulatory receptor signaling on immunometabolism. Additionally, we explore how diet and the microbiome impact immune checkpoint blockade therapy response. The metabolic reprogramming caused by targeting these proteins is essential in understanding immune-related adverse events and therapeutic resistance. This can provide valuable information for potential biomarkers or combination therapy strategies targeting metabolic pathways with immune checkpoint blockade to enhance patient response.

## 1. Introduction

Immune checkpoint proteins are targets of significant interest for cancer therapeutics to enhance T cell antitumor function. Immune checkpoints proteins consist of immunosuppressive and stimulatory signals to regulate self-tolerance and support immune responses [1]. The activation of inhibitory immune checkpoint proteins, such as cytotoxic T-lymphocyte-associated antigen 4 (CTLA-4), programmed cell death protein 1 (PD-1), and programmed death-ligand 1 (PD-L1), impairs the T cell ability to activate, recognize and eliminate cancer cells, allowing uncontrolled cancer growth by bypassing antitumor immune surveillance. Alternatively, activating stimulatory immune checkpoint proteins, like CD28, inducible costimulator (ICOS), glucocorticoid-induced TNFR-related protein (GITR), and 41BB, enhance T cell activation and antitumor function. Several antagonist therapies for inhibitory immune checkpoint protein have been FDA approved for various cancers, while agonist therapies for stimulatory immune checkpoint proteins are still under investigation with several therapies in clinical trials. Although these therapies are revolutionary for cancer treatment, a subset of patients do not respond. Treatment is also frequently complicated by the development of immune-related adverse events (irAE). Therefore, further understanding of immune checkpoint proteins and the impact of their respective targeted therapies is essential to improve therapeutic response and patient survival.

The metabolic state of a T cell is dependent on its respective phenotype and function. Naïve T cells undergo oxidative phosphorylation but shift to aerobic glycolysis when differentiating into cytotoxic (CD8+) T cells [2,3,4,5]. Mitochondria increase in mass and fission while cristae formation decreases to maintain the integrity of CD8+ T cells and their effector function during glycolysis [5,6]. Although aerobic glycolysis is less energy efficient, it produces the necessary precursors to support their high rates of proliferation and cytokine production [2,3,4]. Alternatively, T cells differentiating to a memory phenotype shift to fatty acid oxidation with an increased mitochondrial spare respiratory capacity to support prolonged energy production [7,8]. There is metabolic competition within the tumor microenvironment with T cells, specifically CD8+ T, and cancer cells, as they both undergo aerobic glycolysis, more commonly known as the Warburg effect, to produce energy [9]. Due to nutrient competition created by cancer cells, CD8+ T cells cannot consume the necessary nutrients to sustain their bioenergetic processes, leading to decreased mitochondrial mass, remaining in an inactivated state with an exhausted phenotype and no antitumor cytolytic activity [6]. Therefore, maintaining the integrity of T cell metabolism, specifically in the tumor microenvironment, is essential to their antitumor functionality.

The metabolic reprogramming associated with immune checkpoint proteins is essential to understand the development of resistance and immune-related adverse events (irAE). Furthermore, it can provide valuable information for potential biomarkers or targeted metabolic therapies combined with immune checkpoint therapies to enhance patient response and survival. This review will focus on the metabolic reprogramming of immune and cancer cells due to inhibitory and stimulatory immune checkpoint protein activation and its beneficial and/or detrimental effects on antitumor activity. Additionally, we will explore the impact of diet and the microbiome on the bioenergetics of immune cells and response to immune checkpoint therapies. 

## 2. Metabolic Effects of Inhibitory Immune Checkpoint Protein Activation on Immune Cells

**Figure 1:**
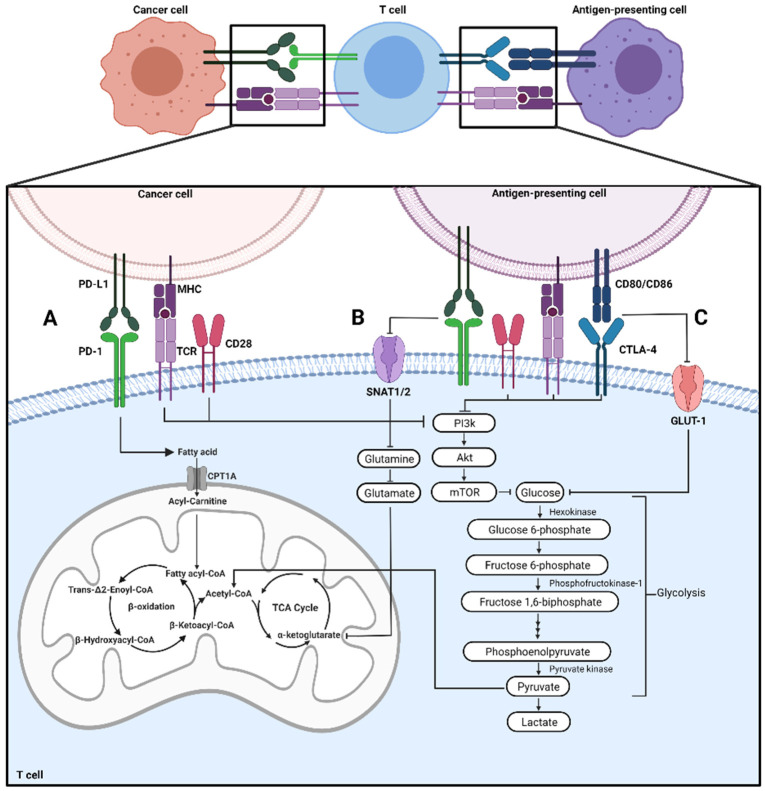
Immune checkpoint proteins regulate metabolic signaling on T cells. (**A**) Interactions with cancer or antigen-presenting cells can modulate T cell metabolism (**B**) PD-L1 binding to PD-1, regulates fatty acid oxidation on T cells and limits glutamine metabolism by reduction of SNAT1/2 (**C**) Activation of CTLA-4 inhibits glycolysis within activated effector T cells inhibiting PI3K/AKT signaling, reduction of glucose uptake by inhibition of GLUT-1.

PD-1 (CD279) and CTLA-4 (CD152) are inhibitory checkpoints of T cell responses [10,11]. PD-1 is often known for its interaction and activation with the ligands PD-L1/PD-L2, while CTLA-4 interacts with the receptor CD80/CD86 (B7-1/B7-2) [11,12,13]. The activation of these inhibitory immune checkpoint proteins alters metabolism within T cells, which can impact their phenotype and respective function (Figure 1). Interestingly, inhibitory immune checkpoint proteins are also expressed and activated on other immune cells, like macrophages and dendritic cells, altering their metabolism and functionality (Figure 1A).

The FDA has developed and approved several therapies to block immune checkpoint proteins. Ipilimumab, a monoclonal antibody that targets CTLA-4, was first approved by the FDA for the use of metastatic melanoma patients [14]. This propelled the development and approval of nivolumab and pembrolizumab, monoclonal antibodies that target PD-1 [12,13]. This section of the review will focus on the metabolic reprogramming associated with activating inhibitory immune checkpoint proteins on both T cells and other immune cells; and how immune checkpoint blockade therapies potentially regulate cell bioenergetics to enhance antitumor immune responses.

### 2.1. Effector T Cells

When PD-1 interacts with PD-L1, the immunoreceptor tyrosine-based switch motif (ITSM) of PD-1 becomes phosphorylated. Src homology region 2 domain-containing phosphatase-2 (SHP2) is recruited to dephosphorylate molecules involved in T cell receptor (TCR) activation [15,16]. Alternatively, CTLA-4 differentially inhibits T cell activation distinct from PD-1 [17]. CTLA-4 has a higher affinity to CD80/CD86 than CD28, the T cell costimulatory receptor, inhibiting TCR signaling [18,19]. Once CTLA-4 is bound to CD80/CD86, CD28 PI3K/Akt signaling is inhibited (Figure 1C) [20,21]. Therefore, both CTLA-4 and PD-1 activation inhibit T cell activation and impair effector T cells cytotoxicity against cancer cells.

Aside from inhibiting activation, PD-1 ligation can alter metabolism within effector T cells. PD-1 ligation on CD4+ T cells inhibits the costimulatory receptor, CD28, from phosphorylating and activating PI3K [17]. Therefore, the necessary PI3K/Akt/mTOR signaling pathway that regulates cellular glycolysis is not activated (Figure 1B) [17]. Expression of transporters that support the cellular intake of glucose are decreased as well as hexokinase activity, these changes are associated with a shift from glycolysis to fatty acid oxidation (Figure 1) [22]. Alternatively, CTLA-4 differentially inhibits effector T cell activation through a separate pathway than PD-1 [17]. Activation of CTLA-4 inhibits glycolysis within activated CD4+ T cells but does not shift the cells to fatty acid oxidation (Figure 1C) [22]. Instead, the cells remain in a quiescent state with little to no activity [22].

In addition to the pathways discussed above, tryptophan metabolism by the enzyme indoleamine 2, 3-dioxygenase 1 (IDO1) may be involved in regulating immune checkpoint proteins. IDO1 conversion of tryptophan to kynurenine is implicated in immunosuppression in the tumor microenvironment and resistance to immune checkpoint blockade therapy [23]. In tumor-infiltrating T cells, kynurenine binding to the aryl hydrocarbon receptor (AHR) regulates PD-1 expression by AHR binding to xenobiotic response element (XRE) motifs in the promoter region of PD-1 [24]. On the other hand, CTLA-4 interaction with CD80/CD86 regulates tryptophan metabolism as a potential mechanism for maintaining peripheral tolerance [25].

Metabolic reprogramming occurring during T cell activation depends on interactions between the endoplasmic reticulum (ER) and mitochondria. These interactions are structurally and functionally modulated through tethering formed at specific subdomains of the ER membrane and mitochondrial-associated membranes (MAMs) [26,27]. This tethering role of the MAMs regulates glucose sensing, lipid synthesis, and rapid release of calcium (Ca^2+^) signals. Disruption of this process impairs activation leading to functionally anergic T cells, which cannot flux Ca^2+^ and activate Nuclear Factor of Activated T cells (NFAT) [28,29,30]. The T cell inability to flux Ca^2+^ properly is also associated with chronic expression of PD-1 [28].

ER stress is implicated in immune checkpoint therapy insensitivity due to cytotoxic T cell dysfunction by several mechanisms, including the recruitment of myeloid suppressive cells, transcription of inhibitory receptors, and metabolic exhaustion [29,30,31,32]. These effects are partly mediated by ER stress canonical activation of the unfolded protein response (UPR) pathway. In particular, PKR-like endoplasmic reticulum kinase (PERK; *EIF2AK3*) activation results in an “exhausted-like” T cell phenotype linked to PD-1 expression in CD8+ T cells [29]. Blockade of PERK alone was associated with increased T cell oxygen consumption rate; furthermore, in vivo blockade of PERK enhanced the antitumor action of anti-PD-1 blockade [29]. This suggests a mechanism to overcome therapeutic resistance to immune checkpoint blockade by re-invigorating T cell bioenergetics.

### 2.2. Exhausted T Cells

Inflammation and chronic antigenic stimulation of T cells from the tumor microenvironment can cause T cells to differentiate into an exhausted phenotype with altered metabolic activity [33]. Exhausted T cells have increased expression of inhibitory receptors like PD-1 and CTLA-4 with decreased effector function and cytokine secretion [33,34,35,36]. The action of PD-1 leads to a decrease in glucose consumption and a decrease in the rate of glycolysis in T cells [31]. In the absence of glucose, short-chain fatty acids such as acetate can serve as carbon sources, and supplementing acetate can restore interferon-gamma (IFNγ) release on exhausted T cells, especially from CD8+ T cells with high expression of PD-1 [37]. This suggests an alternate metabolic pathway involved in response to immune checkpoint inhibition.

Additionally, PD-1 activation decreases PPAR-gamma coactivator 1α (PGC1α), a transcription factor that controls mitochondria biogenesis [6,31]. Therefore, the T cell will have diminished mitochondria biogenesis, function, and ability to undergo oxidative phosphorylation [31]. This overall decrease in bioenergetics by T cells due to PD-1 activation results in the differentiation of an exhausted phenotype. However, by targeting PD-1, AMP-activated protein kinase (AMPK) and mTOR activate, resulting in amplified mitochondrial biogenesis and oxidative phosphorylation due to increased PGC1α expression [38]. This improvement of bioenergetics restores CD8+ T cell activation and proliferation [38].

Metabolic fitness is tightly linked to T cell exhaustion [31]. Terminally exhausted tumor-infiltrating lymphocytes are characterized by accumulation of depolarized mitochondria due to lack of activation of mitophagy [39]. This accumulation of depolarized mitochondria seems to be regulated by TCR and PD-1 signaling [39]. Consistent with these findings, knockout of PD-1 on these cells reduced the population of depolarized mitochondria suggesting that PD-1 signaling regulates mitochondria integrity and cell bioenergetics status. In the same study, in vivo administration of nicotinamide riboside (NAD) improved anti-PD-1 administration suggesting a mechanism to overcome the metabolic insufficiency caused in part by PD-1 signaling.

### 2.3. Memory T Cells

The shift to fatty acid oxidation promotes the differentiation of T cells to a memory phenotype [7,8]. Fatty acid oxidation involves the catabolism of fatty acids to acetyl-CoA, which will subsequently enter the citric acid cycle and oxidative phosphorylation to produce ATP [40]. This type of metabolism supports prolonged survival and rapid expansion [7,8]. When PD-1 is ligated on effector T cells, increased expression of carnitine palmitoyltransferase 1A (CPT1A), the rate-limiting enzyme of fatty acid oxidation, and adipose triacylglycerol lipase (ATGL), an enzyme involved in lipolysis, occurs (Figure 1B) [22]. This promotes lipolysis as indicated by increased free fatty acid and glycerol release [22]. Furthermore, ligation of PD-1 results in the inhibition of amino-acid transport by inhibiting glutamine transporters SNAT1/2 [24]. This prevents metabolism of glutamine through glutaminolysis which is known to support T cell activation [22,41].

Additionally, the mitochondrial spare respiratory capacity of these cells is increased [22], which was observed in the clinic as tissue-resident memory T cells of gastric adenocarcinoma patients undergoing fatty acid oxidation have increased PD-1 expression [42]. CTLA-4 ligation also inhibits glycolysis by inhibiting glucose transporters such as GLUT-1 (Figure 1C). However, CTLA-4 activation does not amplify fatty acid oxidation through CPT1A and ATGL like PD-1 activation, causing T cells to remain in an inactive, quiescent state [22].

Although the longevity of these T cells is enhanced due to increased fatty acid oxidation when PD-1 is activated, the amplification of fatty acid oxidation can result in increased mitochondrial hydrogen peroxide production and total cellular reactive oxygen species (ROS) [43]. This increase in ROS may lead to a decrease in the expression of anti-apoptotic proteins, leading to apoptosis of T cells [43,44].

### 2.4. Macrophage and Dendritic Cells

Macrophage plasticity is associated with metabolic reprogramming. While macrophage polarization is defined in a complex spectrum of differentiation, M1 macrophages tend to favor glycolysis, whereas M2 macrophages tend to favor oxidative phosphorylation [45]. Inhibitory immune checkpoint proteins are expressed on other immune cells aside from T cells [42,43]. It is reported that PD-1 and PD-L1 are expressed on macrophages; some reports suggest that PD-L1 expression is increased in the M2-like phenotype [46,47]. Whether the expression of these checkpoints is linked to metabolic reprogramming on macrophages is not well understood. Within a hypoxic tumor microenvironment, PD-L1 expression on macrophages and dendritic cells is mediated by the activation of the M2 isoform of pyruvate kinase (PKM2) [44]. Pyruvate kinase is the final glycolysis enzyme that converts phosphoenolpyruvic acid to pyruvate, with PKM2 associated with tumor progression and metabolic reprogramming [45].

Additionally, ROS have been shown to upregulate PD-L1 expression on macrophages, resulting in immunosuppressive activity [48]. Lipid metabolism and ROS production are implicated in the immunosuppressive activity of myeloid-derived suppressor cells (MDSCs) and response to anti-PD-L1. In two preclinical mouse models, inhibiting fatty acid transport protein 2 (FATP2) enhanced PD-L1 blockade, which was due to a reduction in lipid accumulation and ROS production. Additionally, there was a decrease in expression of PD-L1 and an increase in CD107a [48], which is also known as lysosomal-associated membrane protein-1 (LAMP-1) [49].

Alterations in UPR signaling can also impact immune checkpoint therapies due to alterations in macrophage polarity. Melanoma patients who are non-responsive to anti-CTLA-4 therapy have increased circulation of pro-tumorigenic and immunosuppressive M2-like macrophages (CD206+) with increased UPR signaling [46]. This potentially impairs the ability of the patient to respond to immune checkpoint blockade therapy, resulting in disease progression [46]. Therefore, treating patients with immune checkpoint blockade influences T cell and other cell types metabolic signaling within the tumor microenvironment, potentially impacting therapeutic responsiveness and resistance.

## 3. Metabolic Effects of Stimulatory Immune Checkpoint Protein Activation on Immune Cells

The activation of stimulatory immune checkpoint proteins plays essential roles in T cell metabolism, proliferation, cytokine secretion, and survival [1]. Several clinical trials have been or are currently being performed to target stimulatory immune checkpoint proteins to promote antitumor responses and reduce tumor burden for various cancer patients [50]. While there are several stimulatory immune checkpoint proteins in this review, we focus on CD28, ICOS, GITR, and 4-1BB due to the effects on metabolism and clinical stage of agonist drugs.

### 3.1. CD28

**Figure 2:**
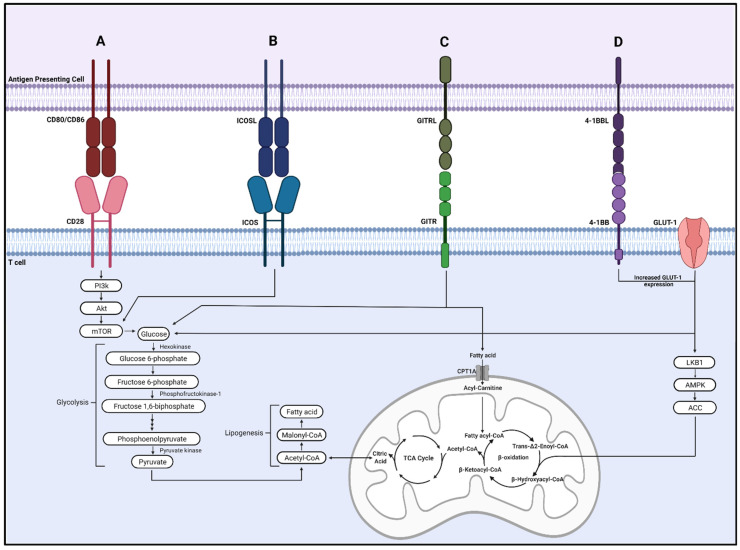
Stimulatory checkpoint proteins regulate metabolic signaling. (**A**) CD28 activation promotes PI3K/Akt/mTORC1 pathway signaling, increasing glycolysis and mitochondrial metabolism. (**B**) ICOS/ICOSL activation increases glucose uptake and metabolism through mTOR activation. (**C**) GITR activation can stimulate the TCA cycle by metabolism lipid, glucose, and other nutrient stores (**D**) 4-1BB activation increases GLUT-1 expression to enhance glycolysis and fatty acid metabolism through LKB1/AMPK/ACC pathway activation.

CD28 is a widely studied costimulatory receptor that recognizes CD80/CD86 on antigen-presenting cells [51]. As a critical regulator of T cell metabolism, CD28 promotes PI3K/Akt/mTORC1 pathway activation to increase glucose and mitochondrial metabolism, resulting in increased T cell proliferation and effector function (Figure 2A) [52].

As the CD28-CD80/CD86 signaling axis is a main stimulatory immune checkpoint, great interest was given to modulating its activity using a superagonist monoclonal antibody to increase T cell function for the treatment of autoimmune, inflammatory diseases, and cancer [53]. Preclinical studies in mice and rats using TGN1412 (CD28 superagonist) showed promising results; however, in a Phase I clinical trial, all six healthy volunteers experienced a life-threatening systemic release of proinflammatory cytokines, commonly referred to as a cytokine storm [54]. Due to the unexpected outcome, clinical trials involving the CD28 superagonist were immediately terminated [55].

### 3.2. Inducible Costimulator (ICOS)

Although targeting CD28 resulted in severe side effects, several other stimulatory immune checkpoint proteins are of interest. They promote immune function through alterations of metabolic processes and show promising preclinical results for cancer treatment. ICOS is a member of the CD28 family of costimulatory molecules and is upregulated on the surface of T cells following T cell activation and upon binding to its ligand (ICOSL) on antigen-presenting cells [56]. ICOS promotes T cell proliferation, T helper 2 (Th2) differentiation and directly modulates metabolism [57]. During T follicular helper (Tfh) cell differentiation, ICOS ligation promoted glucose uptake and metabolism through mTOR activation [58]. ICOS activated mTORC1/mTORC2 to drive glucose transporter-1 (GLUT-1) mediated glucose metabolism and lipogenesis to promote Tfh cell response (Figure 2B) [59]. Deficiency of mTORC1/mTORC2 reversed these effects and impaired CD4+ T cell accumulation and immunoglobulin A production [59].

There are currently two clinical trials studying the drug GSK3359609, an ICOS receptor agonist antibody, intended to treat solid tumor cancers [60,61]. The first clinical trial is a Phase I dose-escalation and expansion study to investigate the safety, pharmacology, and preliminary antitumor activity for patients with advanced solid tumors [60]. The second clinical trial is a Phase II study conducted in non-small cell lung cancer patients. This trial aims to compare the clinical activity of novel regimens, including GSK3359609, in combination or as a single agent for the standard of care for non-small cell lung cancer patients [61]. Regarding safety, preliminary results report that the safety profile of GSK3359609 in combination with chemotherapy is manageable as most adverse events were grades 1 or 2 and consistent with known chemotherapy toxicities [62].

### 3.3. Glucocorticoid-Induced TNFR-Related Protein (GITR)

**Figure 3:**
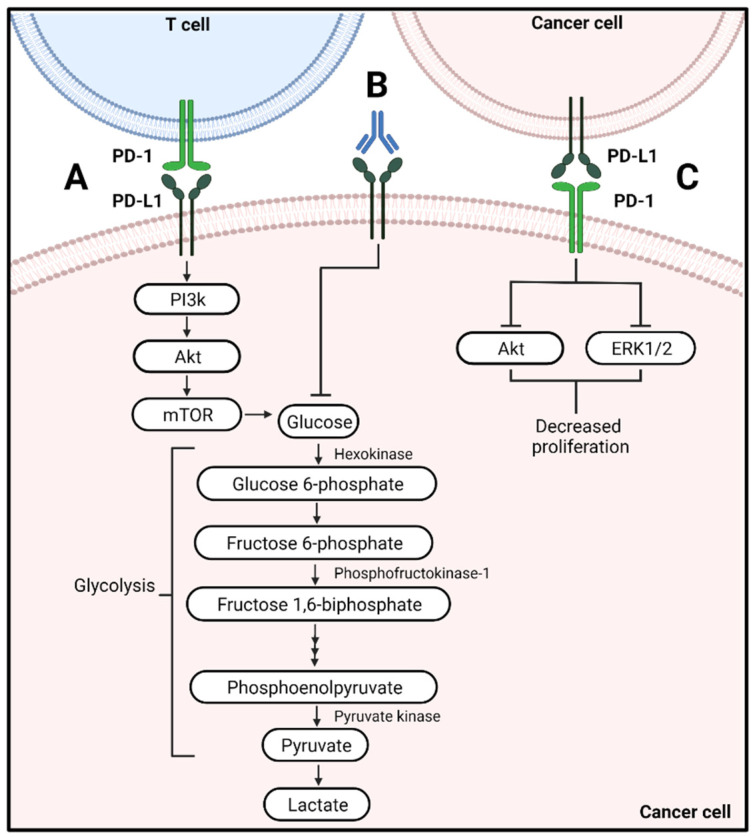
Regulation of metabolic signaling of immune checkpoint inhibitors on cancer cells. (**A**) PD-L1 engagement results in activation of glycolysis and activation of the PI3K/Akt/mTOR pathway. (**B**) Antibodies to PD-L1 are known to inhibit its metabolic signaling. (**C**) PD-1 on cancer cells limits Akt and ERK1/2 signaling regulating cancer cell proliferation.

GITR is a member of the tumor necrosis factor receptor (TNFR) family highly expressed on T regulatory (Treg) cells and present on effector T lymphocytes, natural killer cells, and neutrophils [63,64]. GITR is activated by its ligand, GITRL, mainly expressed on antigen-presenting and endothelial cells [65]. GITR activation on effector T cells generates a positive costimulatory signal and promotes T cell activation and proliferation; however, the activation of GITR on Treg cells abolishes their suppressive function in cancer settings [66]. GITR agonists increase metabolism to support CD8+ T cell proliferation and effector function by upregulating nutrient uptake, lipid stores, glycolysis, and oxygen consumption rate (Figure 3C) [67]. Although GITR stimulation primarily enhances the proliferation of Treg cells, several studies suggest that may not be the case in the context of cancer; instead, GITR agonists reduce tumor growth by increasing effector T cells and reducing Treg cells [68,69,70].

A Phase I/II clinical trial is currently in progress to determine possible side effects and optimal dose of a GITR agonistic monoclonal antibody (BMS-986156). In addition, BMS-986156 will be given together with ipilimumab and nivolumab, with or without stereotactic body radiation, to evaluate efficacy in patients with lung/chest or liver cancer that has metastasized [71].

### 3.4. 4-1BB

4-1BB/CD137 is a member of the TNFR family of costimulatory receptors and is expressed on activated CD4+ and CD8+ T cells [72]. 4-1BB acts as a potent costimulator of T cells, increasing T cell proliferation and expansion and promoting a memory-like phenotype [73]. Additionally, several studies show that 4-1BB agonists promote T cell metabolic reprogramming. Choi et al. demonstrated that a 4-1BB agonist increased T cell glucose and fatty acid metabolism, in part by enhancing GLUT-1 expression and activating the liver kinase B1 (LKB1)/AMPK/acetyl-CoA carboxylase (ACC) signaling pathway (Figure 2D) [74]. Menk et al. found that 4-1BB costimulation enhanced CD8+ T cell mitochondrial capacity and increased transcription of energy metabolism genes by activating p38-MAPK [75].

Currently, CD19-targeted chimeric antigen receptor T cells containing a 4-1BB costimulatory domain are FDA approved to treat B-cell pediatric leukemia and refractory B-cell lymphoma [76]. However, over 15 active or recruiting clinical trials are investigating using 4-1BB agonistic monoclonal antibodies in cancer patients [77]. Initial trials using urelumab showed promising results as a single agent in patients with advanced cancer. However, trials had to be terminated because of severe adverse effects, including liver inflammation [78]. Due to previous results, current studies have drastically reduced the dose and evaluated urelumab effects in combination with other immune-modulating agents [77]. In contrast, utolimumab has no dose-limiting toxicities and shows preliminary antitumor activity in patients with advanced cancer; however, utolimumab is a weak 4-1BB agonist with little efficacy as a monotherapy and is now in clinical trials involving combination therapy to increase effectiveness [79]. Phase 1 clinical trials combining utolimumab with rituximab in patients with relapsed or refractory non-Hodgkin’s lymphoma and utolimumab with pembrolizumab in patients with advanced solid tumors have demonstrated tolerability, safety, and preliminary clinical activity [79,80].

## 4. Metabolic Effects of Immune Checkpoint Protein Activation on Cancer Cells

Cancer cells often overexpress the immune checkpoint protein PD-L1 (CD274). PD-L1 is commonly known for its interaction with PD-1, limiting T cell antitumor function as discussed above. However, PD-L1 can also alter the metabolism of cancer cells and nutrient availability in the tumor microenvironment, impacting T cell antitumor function. Atezolizumab, avelumab, and durvalumab are FDA-approved monoclonal antibodies that target PD-L1 for various cancers, including melanoma, non-small cell lung cancer, urothelial carcinoma, and metastatic triple-negative breast cancer [81,82,83]. Additionally, it has recently been discovered that cancer cells can also express PD-1, resulting in the reprogramming of cancer cell metabolism [84].

### 4.1. Metabolic Alterations of Cancer Cells Associated with PD-L1 Signaling

Cancer cells undergo aerobic glycolysis as their primary energy source, resulting in increased glucose uptake and depletion of glucose from the tumor microenvironment [85]. When PD-L1 was targeted with a monoclonal antibody, a decrease in glycolysis was observed in various cancer cells (Figure 3A,B) [86]. This is due to the inactivation of the PI3K/Akt/mTOR pathway and the translation of glycolytic enzymes that regulate glycolysis within these cells [86]. The decrease in glycolysis by the cancer cell may result in increased availability of glucose in the tumor microenvironment that can be used by T cells to sustain their effector phenotype and antitumor function [86].

Due to the high proliferation rate by cancer cells, an abundance of lipid biomass precursors is essential to support the formation of membranes of newly proliferating cells. An integral component of membranes is phosphatidylcholine, resulting in cancer cells’ overexpression of choline kinase alpha (Chk-α) [87]. Chk-α is an enzyme that phosphorylates choline to phosphocholine, subsequently increasing choline metabolism and the availability of choline-containing compounds. PD-L1 and Chk-α have been shown to have an inverse relationship that impacts lipid metabolism and immunosuppression [88]. Targeting PD-L1 increased phosphocholine due to the increase in Chk-α, potentially providing precursors to support proliferation through membrane formation [88]. However, when PD-L1 and Chk-α are targeted in combination, these changes were attenuated as no metabolic changes were observed [88]. Therefore, PD-L1 regulates cancer cell metabolism through Chk-α, and targeting both PD-L1 and Chk-α in combination may be beneficial to enhance antitumor immune cell surveillance to improve response to immune checkpoint blockade therapy.

In another set of studies, PD-L1 blockade was associated with lipid peroxidation in tumors, increasing ferroptosis [89,90]. Ferroptosis is a form of cell death characterized by activation of iron-dependent lipid peroxidation [90]. The mechanism implicated IFNγ release from CD8+ T cells which downregulates *SLC3A2* which mediates the exchange of extracellular cystine and intracellular glutamate. In the study, treatment with anti-PD-L1 resulted in limitation of cancer cell cysteine uptake and glutamate release, subsequently stimulating lipid peroxidation and ferroptosis [90]. The activation of ferroptosis was linked to enhanced CD8+ T cell ant-tumor immunity resulting in better tumor clearance with immune checkpoint blockade [90].

The observed upregulation of PD-L1 under hypoxia can also result in its translocation to the nucleus resulting in a switch from apoptosis to pyroptosis in cancer cells [91]. Pyroptosis is a form of cell death characterized by the gasdermin C (GSDMC) mediated cleavage of caspases [91]. In hypoxic conditions, p-Stat3 interacts with PD-L1, facilitating translocation to the nucleus and subsequently enhancing GSDMC transcription [91]. In macrophages, metabolites of the TCA cycle, such as fumarate, regulate pyroptotic cell death. Experiments show that dimethyl fumarate causes the succination of gasdermin D [92], preventing its interaction with caspases limiting pyroptotic cell death [92]. While it is unknown that PD-L1 directly regulates this process, the succination of gasdermin could play a potential role in response to anti-PD-L1 therapy.

Additionally, several immunosuppressive metabolites like lactate, glutamate, S-methyl-5′-this adenosine (MTA), or glutamine were increased when PD-L1 was targeted along with the cytokines Transforming growth factor-β (TGF-β) and Cyclooxigenase-2 (COX-2) [88]. These metabolites have previously been examined to impair immune cell antitumor function in the tumor microenvironment [93,94,95,96].

### 4.2. Metabolic Alterations by PD-1 on Cancer Cells

A lesser-known aspect of PD-1 is its expression on cancer cells. In over thirty types of cancer, the gene that encodes for PD-1, *PDCD1*, has been found [84]. PD-1 expression on these cancer cells may suppress proliferation as a decrease in Akt and ERK1/2 activity was observed (Figure 3C) [84]. Therefore, when therapies were delivered to target PD-1, enhanced cancer cell growth was observed as Akt, and ERK1/2 activity was no longer suppressed [84]. It is evident from these studies the impact the activation and blockade of PD-1 can have on both T cells and cancer cells.

### 4.3. Regulation of PD-L1 Expression by Metabolic Pathways

**Figure 4:**
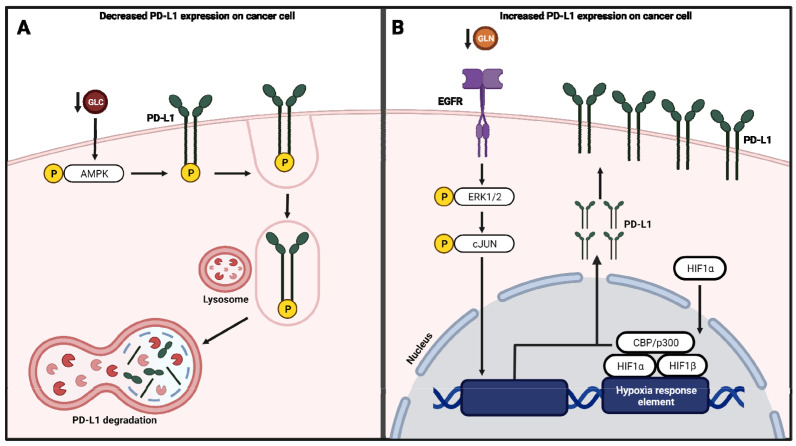
Metabolic signaling and regulation of PD-L1 expression. (**A**) Glucose (GLU) starvation and activation of AMPK results in the degradation of PD-L1 and a decrease in its expression. (**B**) Glutamine (GLN) depletion activates EGFR and MAPK signaling, resulting in the upregulation of PD-L1 during hypoxia.

The protein level expression of PD-L1 in cancer cells seems to be regulated by various factors (Figure 4). PD-L1 protein levels (but not mRNA) were downregulated in human and mouse cancer cells subjected to glucose starvation which was linked to activation of AMPK (Figure 4A) [97]. Furthermore, AMPK phosphorylation of PD-L1 results in its degradation by autophagy. In the same study, inhibition of glycolysis with 2-DG resulted in the activation of AMPK and downregulation of PD-L1 [97].

In vitro and in vivo studies using renal cancer cell lines have indicated that glutamine depletion upregulates PD-L1 protein expression (Figure 4B) [98]. This is due to the activation of EGFR signaling through the mitogen-activated protein (MAP) kinase signaling. Inhibitors of EGFR and proteins of the MAP kinase protein ERK and C-Jun downregulated PD-L1 expression suggest that PD-L1 expression is subjected to regulation by this pathway [98]. Furthermore, it is reported that fatty acid synthase (FASN) expression in a leukemia cell line is linked to an increase in PD-L1 expression, suggesting an immunosuppressive role of fatty acid synthesis [99]. Orlistat treatment, which decreases FASN, decreased PD-L1 expression [99]. Thus, suggesting a potential strategy to overcome immunosuppression and overcome resistance to immune checkpoint therapies.

Hypoxia is a common characteristic amongst solid tumors due to their tortuous vasculature and increased oxygen consumption by cancer cells undergoing aerobic glycolysis [100]. Cancer cells must adapt to the hypoxic tumor microenvironment to sustain metabolic processes to support their high proliferation rates and survival by shifting from the oxygen-dependent mitochondrial oxidative phosphorylation to glycolysis [101]. Previous data has shown that patients with hypoxic tumors often have low response rates to immune checkpoint blockade therapies and decreased overall survival [102,103,104,105]. This may be due to impaired T cell activation, proliferation, cytokine production, and cytolytic capacity from the lack of oxygen [106,107,108,109,110]. Therefore, improving oxygenation within tumors may enhance immune checkpoint blockade therapy response. Cancer cells also stabilize and accumulate hypoxic responsive factors (HIF) within the tumor microenvironment, which will bind to hypoxia response elements (HRE) in the promoter region of hypoxia-responsive genes like PD-L1 (Figure 4B) [111,112]. With the upregulation of PD-L1 expression on cancer cells, T cell effector function inhibition can occur due to PD-1 interaction [112,113]. Therefore, hypoxia may be a marker of immune checkpoint blockade response, and the oxidative metabolism of tumors impacts the ability of T cells to respond to immune checkpoint blockade therapy due to increased PD-L1 expression.

## 5. Impact of Diet and the Microbiome on Immune Checkpoint Blockade Response

Dietary intake impacts host metabolism and thus is implicated in carcinogenesis. Dietary intake, obesity, and microbial-derived metabolites can alter immune checkpoint protein expression, immune cell metabolism, and subsequent response to immune checkpoint blockade. Therefore, the role of diet and its impact on immune checkpoint signaling remains an active area of study to improve outcomes in cancer patients.

### 5.1. Dietary Interventions

Several dietary interventions have been examined to test response to immune checkpoint blockade. A high fiber diet is implicated in immune checkpoint response regulation. This may be partly due to the bacterial processing of fiber and the production of short-chain fatty acids such as acetate and butyrate that, as discussed above, influence immune cell differentiation and function [114]. The ketogenic diet is associated with anticancer effects, specifically by enhancing antitumor immunosurveillance by reducing the percentage of CD8+ T cells positive for PD-1, CTLA-4, and reducing PD-L1 expression on tumor cells [115]. It is reported that mice fed a ketogenic diet have better responses to anti-PD-1 therapy. In the same study, feeding mice 3-hydroxybutyrate caused a similar anticancer effect associated with ketogenic diet consumption. In vivo T cell depletion experiments in mice reversed the antitumor effects of a ketogenic diet, implicating that the metabolic effect of diet enhanced antitumor immunosurveillance due in part to immune checkpoint protein expression regulated by diet [116]. Other studies have found that the ketogenic diet was associated with downregulated PD-L1 protein levels in a murine model of colorectal cancer. In vitro experiments in low-glucose culture media supplemented with the ketone, body β-hydroxybutyrate demonstrated that the changes in PD-L1 expression were not regulated by ketone body supplementation but were due to the low levels of glucose in media [97]. Furthermore, the mechanism for PD-L1 downregulation was mediated by AMPK phosphorylation of PD-L1 and subsequent degradation by autophagy [97].

Caloric restriction diets are also implicated in response to immune checkpoint therapy. In an animal study, caloric restriction by fasting or using caloric restriction mimetics enhanced anti-PD-1/PD-L1 antibody response when combined with agents known to induce immunogenic cell death [117]. It is thought that fasting or caloric restriction mimetics can facilitate the release of ATP from dying cells, which stimulates an innate antitumor immune response. However, calorie restriction also was shown to affect tumor-infiltrating lymphocytes, which is characterized by the reduction in nucleocystosolic acetyl CoA and activation of autophagy. This results in a signaling cascade that increases T cell stemness resulting in enhanced antitumor immunity [118].

### 5.2. Obesity

Prolonged over-nutrition can cause obesity with anatomic and functional abnormalities in adipose tissue leading to altered metabolic homeostasis and adipokine production [119,120,121]. The link between obesity is the progression of several types of cancer is well established; however, clinical studies show a paradoxical relationship between obesity and immune checkpoint blockade response [122]. In a study of metastatic melanoma treated with targeted immune checkpoint blockade, obese patients were found to have improved progression-free and overall survival compared to non-obese patients [123]. These observations have been primarily observed in male patients with a non-specific link in female patients [123]. In a preclinical study of breast cancer, the anti-PD-1 treatment effect on tumor growth was augmented in obese mice compared to lean [122]. This study found that this might be partly due to increased microbiome diversity, consistent with clinical studies examining microbiota influence on immune checkpoint therapy response [122,124]. While the mechanisms remain to be elucidated, it is evident that host metabolism and microbiota influence immune checkpoint blockade therapeutic responsiveness.

### 5.3. Microbiome

While highly dependent on geographical location, cancer type, and treatment type, studies analyzing the gut microbiome populations of patients prior to immune checkpoint blockade initiation have correlated microbes with responsiveness identifying a consortium of immunotherapy responder associated microbes (Table 1) [124,125,126,127,128]. Suggesting the possibility of microbiota-based therapeutic strategies to modify immune checkpoint signaling and responsiveness.

Studies of psoriasiform dermatitis in mice suggest that feeding a Western diet increases PD-1 expression on gamma-delta low T cells in the skin compared to control diet-fed mice [129]. Another mechanism by which diet could regulate immune checkpoint protein signaling is modulation of the microbiome. Investigating immune checkpoint blockade response mediators in different cancer types shows that the gut microbiome regulates immune checkpoint blockade responsiveness by pre-programming the immune system. The elevated probiotic or commensal gut microbiome is associated with PD-1 responsiveness in numerous cancer types; *Prevotella* in gastrointestinal cancer, *Akkermansia muciniphila* with non-small cell lung cancer, and renal cell carcinoma, *Bifidobacterium longum* in non-small cell lung cancer, and *Lactobacillus* metastatic melanoma [128,129,130,131,132].

Whether there is a direct link between specific microbial species-mediated immune checkpoint protein expression regulation remains to be explored; however, it is known that the microbiota can regulate tryptophan processing [133]. As discussed above, the binding of tryptophan metabolites to the AHR receptor can regulate PD-1 expression. Therefore, the regulation of tryptophan by microorganisms and its effect on metabolism could link to immune checkpoint protein expression regulation.

**Table 1:** Clinical studies investigating gut microbiome correlations with immune checkpoint blockade therapy responsiveness. Cancers: NSCLC: non-small cell lung carcinoma, RCC: renal cell carcinoma, GI: gastrointestinal. Treatments: I: Ipilimumab, N: Nivolumab, P: Pembrolizumab, A: Atezolizomab.

Cancer	Study Size	Geographical Location	Treatment	Microbiota Associated with Favorable Response	Reference
Melanoma	N = 39	Texas, USA	I, N, I+N, P (all) IN P	*B. caccae* *F. prausnitzii, B. thetaiotamicron* *D. formicogenerans*	Frankel et al., 2017 [126]
NSCLC and RCC	N = 100	Paris, France	N	*Akkermansia muciniphilia*	Routy et al., 2018 [125]
Melanoma	N = 42	Illinois, USA	I, N, or P	*Bifidobacterium longum, Collinsella aerofaciens, and Enterococcus faecium*	Matson et al., 2018 [127]
Melanoma	N = 53	Texas, USA	N or P	*Faecalibacterium prausnitzii*	Gopalakrishnan et al., 2018 [124]
NSCLC	N = 37	Shanghai, China	N	*Alistipes putredinis, Bifidobacterium longum*, and *Prevotella copri*	Jin et al., 2019 [130]
GI	N = 74	Beijing, China	I, N, P, or A	*Prevotella/Bacteroides* ratio, *Lactobacillus,* *Akkermansia muciniphilia*	Peng et al., 2020 [128]

## 6. Metabolism and Immune-Related Adverse Events (irAEs) Associated with Immune Checkpoint Blockade Therapy

The role of immune cell metabolism and the onset of irAE due to immune checkpoint blockade therapy is currently under investigation; however, the potential role of bioenergetics and irAE can be extrapolated from studies in autoimmune diseases. Autoimmune disease is characterized by dysregulation of the immune system and an immune response against autologous tissues due to a lack of self-tolerance. Blocking the pathway associated with immune checkpoint proteins can lead to impaired tolerance and the development of autoimmunity. The role of PD-1/PD-L1 in peripheral tolerance was first noted in PD-1 deficient mice that developed autoimmunity, including lupus-like symptoms and immune-mediated dilated cardiomyopathy [131,134].

T helper 17 (Th17) cells are a unique lineage of proinflammatory effector and memory T helper cells that play a prominent role in the pathogenesis of systemic lupus erythematosus (SLE), rheumatoid arthritis (RA), psoriasis, multiple sclerosis (MS), and inflammatory bowel disease (IBD) by activation of pathogenic CD4+ and CD8+ T cells [132,135,136,137]. As described earlier, effector T cells are dependent on nutrients derived from the microenvironment. T cells for these diseases have a unique altered T cell metabolic signature systemically and locally at the tissue level [138,139,140]. Glycolysis is a major metabolic pathway involved in Th17 responses that serve as a metabolic checkpoint for Th17 and Treg cell differentiation. When glycolysis is inhibited, Th17 development is decreased, and the growth of Treg cells is promoted [141]. In an experimental autoimmune encephalomyelitis MS mouse model, an increase in degradation of GLUT-1 containing vesicles was shown to decrease the severity of encephalomyelitis [142]. T cells from patients with SLE have increased mitochondria fission, which functions to mitigate mitochondrial stress and reduce the creation of new mitochondria through mitochondrial fusion [143]. In addition, T cells have increased oxidative phosphorylation and aerobic glycolysis associated with producing excess levels of ROS and defective lipid metabolism [144]. On the same line, hypoxia is known to regulate differentiation of Th17 lineage and Treg cells, where the lack of HIF-1 was associated with increased gene expression of CTLA-4 [141]. Altered lipid metabolism and serum total fatty acid profile changes are observed in individuals with autoimmune disease [145,146]. Although it is unclear how a modified lipid profile promotes autoimmunity, long-chain fatty acids have been shown to enhance the differentiation of Th1 and Th17 cells [147]. Patients who consume low-fat diets and are treated with statins have a decrease in the severity of the autoimmune disease [148].

There are several cases of autoimmune diabetes with the treatment of immune checkpoint inhibitors. It is reported that hyperglycemia is associated with checkpoint blockade and can occur in up to approximately 5% of treated patients [149]. Furthermore, in patients with diabetes, the use of immune checkpoint blockades can worsen glucose control, in particular, PD-1/PD-L1 therapies, as PD-L1 is expressed on pancreatic beta cells. While the onset of hyperglycemia may not require treatment cessation, in some cases, immune checkpoint blockade can result in rapid ketosis. Thus, monitoring glucose levels in patients undergoing treatment is warranted [149]. These studies begin to provide insight into irAE that can develop due to immune checkpoint blockade therapy for patients with autoimmune disease, providing a foundation to continue to examine alternative treatment regimens and treatments to reduce irAE for cancer patients.

## 7. Clinical Perspective of Immune Checkpoint Blockade Therapy

One of the top challenges in cancer immunotherapy is “understanding the molecular and cellular drivers of primary vs. secondary immune escape to checkpoint blockade therapies” [150]. This includes understanding how metabolic signaling in the tumor microenvironment impacts immune checkpoint protein expression and whether these proteins induce signaling to regulate cellular metabolism. This could potentially lead to finding targetable options to potentiate immune checkpoint blockade or enhance costimulatory receptor signaling to increase therapeutic responsiveness.

Preclinical and clinical trials have identified metabolic targeting drugs with the potential to enhance the efficacy of immune checkpoint blockade therapy [151,152]. The synergism of immune checkpoint blockade therapy combined with metformin against tumor burden has been demonstrated in preclinical studies [153,154,155]. This is potentially due to the inhibition of the oxygen consumption of tumor cells, which would reduce hypoxia in the tumor microenvironment [156]. Presumably, this reduction in hypoxia would shift nutrient availability in the tumor microenvironment facilitating metabolic reprogramming of T cells and response to immune checkpoint blockade therapies [151].

Some trials have revealed encouraging results using metformin and immune checkpoint blockade. In a trial of 55 metastatic melanoma patients, a cohort of patients treated with ipilimumab, nivolumab, and or pembrolizumab combined with metformin had a 68.2% overall response rate when compared to 54.5% overall response rate in patients with the same immune checkpoint blockade treatment without metformin [157]. Furthermore, an increase in overall survival of 46.7 vs. 28 months and progression-free survival of 19.8 vs. 5 months were observed with the addition of metformin to the immune checkpoint blockade regimen; however, these results did not reach statistical significance, possibly due to small sample size [157]. Not all studies have demonstrated enhanced antitumor activity. A meta-analysis of clinical trials reported that the addition of metformin to immune checkpoint blockade regimens did not impact patients’ overall and progression-free survival [153]. The antitumor and immune effects of metformin are complex. Dosing and scheduling can have profound effects, and the challenges lie in determining optimal treatment regimens [158]. Furthermore, the impact of metformin on immune checkpoint protein expression should be further elucidated as studies have shown that metformin inhibits PD-L1 by endoplasmic reticulum-associated protein degradation in breast cancer cells [154].

As discussed earlier, the activation of IDO is critical in regulating immunosuppression in the tumor microenvironment. Small molecule inhibitors to IDO aim to halt the conversion of tryptophan to the immunosuppressive metabolite kynurenine, which have been studied in clinical trials in combination with immune checkpoint blockade therapy with mixed results [155]. Additionally, ATP release from chemotherapy-treated leukemia cells is shown to induce IDO-1, causing increases in Tregs and tolerogenic dendritic cells that can limit response to immune checkpoint blockade [159].

Phase I/II clinical trials with the IDO inhibitor, epacadostat, in combination with pembrolizumab showed encouraging results [160]; however, the combination treatment failed to improve in phase III clinical trial pembrolizumab efficacy [161]. Another IDO inhibitor, indoximod, showed a modest objective response rate in combination with PD-L1 in melanoma patients compared to treatment alone. Indoximod acts downstream of IDO1 to stimulate mTORC1, possibly acting through a different mechanism from other IDO inhibitors [162]. Further studies are needed to test the efficacy of IDO modulators in combination with immune checkpoint blockade therapy.

Another challenge is finding biomarkers of response, secondary resistance, and toxicity to guide treatment decisions. Metabolic determinants, including the IDO pathway, may prove helpful as a biomarker of immune checkpoint blockade therapy response. Clinical studies have found that the ratio of kynurenine to tryptophan in serum is associated with therapeutic resistance and worst survival to nivolumab [23]. Microbiota-derived short-chain fatty acid acids such as butyrate have been shown to limit CTLA-4 response in mouse models [163]. In the same study, patients who responded to anti-CTLA-4 therapy had low butyrate concentrations at baseline and higher CD4+ memory T cells than patients with elevated serum butyrate [163]. This data suggests that circulating metabolites could be useful in predicting responses to immune checkpoint blockade. Whether that is the case remains to be seen.

It is evident from the studies discussed above that the activation of both inhibitory and stimulatory immune checkpoint proteins can have a drastic alteration to both immune and cancer cell bioenergetics. This metabolic reprogramming can enhance or impair immune cell antitumor function and disease progression. Although antagonist and agonist therapies have been created to target immune checkpoint proteins and restore antitumor immune cell metabolism and function, several factors like diet and the microbiome can impact patient therapeutic response. Therefore, further research is needed to determine biomarkers and other targetable therapeutics combined with immune checkpoint therapies to improve patient response and survival.

## Data Availability

Not applicable.
